# An efficient machine learning framework to identify important clinical features associated with pulmonary embolism

**DOI:** 10.1371/journal.pone.0292185

**Published:** 2023-09-28

**Authors:** Baiming Zou, Fei Zou, Jianwen Cai

**Affiliations:** 1 Department of Biostatistics, University of North Carolina at Chapel Hill, Chapel Hill, NC, United States of America; 2 School of Nursing, University of North Carolina at Chapel Hill, Chapel Hill, NC, United States of America; University of Pisa, ITALY

## Abstract

A misdiagnosis of pulmonary embolism (PE) can have severe consequences such as disability or death. It’s crucial to accurately identify key clinical features of PE in clinical practice to promptly identify potential PE patients who may present asymptomatically, and to prevent misdiagnosing PE as asthma exacerbation in patients with symptoms like dyspnea or chest pain. However, reliably identifying these important features can be challenging due to many factors influencing the likelihood of PE development in complex fashions (e.g., the interactions among these factors). To address this difficulty, we presented an effective framework using the deep neural network (DNN) model and the permutation-based feature importance test (PermFIT) procedure, i.e., PermFIT-DNN. We applied the PermFIT-DNN framework to the analysis of data from a PE study for asthma exacerbation patients. Our analysis results show that the PermFIT-DNN framework can robustly identify key features for classifying PE status. The important features identified can also aid in accurately predicting the PE risk.

## 1 Introduction

Pulmonary embolism (PE) is a blockage in one of the arteries in lung that can occur in an artery in the centre or near the edge of lung. Frequently, PE results from a blood clot that forms in the legs or other part of the body and travels to the lung [1–3]. PE can cause pulmonary hypertension [4–8], and it is associated with the risk of major bleeding [9–12]. Untreated PE can potentially lead to fatal conditions such as chronic pulmonary hypertension, fairly acute right heart failure, permanent damage to the lung, disability or even death. A false diagnosis thus exposes patients to unnecessary risk of complications from PE [13–16]. Clinically, PE may present with conventional symptoms such as shortness of breath, coughing up blood and pleuritic chest pain, dyspnea, but also other symptoms, for examples, insidious onset of breathlessness over days or weeks, or syncope with relatively few respiratory symptoms. On the other hand, many people who have pulmonary embolism do not show any symptoms. The signs and symptoms of PE vary greatly depending on the size of the clot, how much of the lung is involved and whether the patient has an underlying medical condition [17–20].

In addition to the symptoms and clinical presentations heterogeneity of PE, there are many factors associated with the risk of developing PE. Studies have shown that the major factors contributing to an increase in risk of development of pulmonary embolism include heart disease, certain types of cancer, obesity, acute paraplegia, accidental and operative trauma, air travel, inactivity, smoking, etc [21–26]. Even more challenging, these clinical features and symptoms interact with each other and jointly influence the propensity of developing PE in a complex fashion. Identifying important clinical features associated with PE status is helpful to identify many potential PE patients who do not show any symptoms. This will conveniently raise prompt warnings for physicians and patients such that the exact PE status can be timely confirmed by the computed tomographic pulmonary angiography (CTA) to make appropriate treatment decisions. Furthermore, identifying important clinical features of PE can help to avoid diagnosing PE as asthma exacerbation in cases with symptoms such as dyspnea or chest pain [27]. However, it is challenging to determine the key clinical features due to the complex functional relationship between risk features and PE phenotype. On the other hand, many machine learning methods allow robustly modeling the complex relationship between disease outcomes and clinical features. In particular, the deep learning method, e.g., deep neural network (DNN) [28, 29], is a powerful machine learning tool that can accurately approximate a complex functional relationship between disease outcomes and clinical features [30]. Indeed, some machine learning methods such as support vector machine and random forest, etc, have been used for PE predictions [31] but not for the use of identifying critical features associated with PE status.

Though machine learning models can offer the optimal prediction power under complex functional structures, they suffer from the lack of transparency for interpreting each feature’s role in disease outcome prediction due to the abstract algorithm used. How to robustly identify the important clinical features associated with PE risk is critical yet not trivial. An existing procedure for this purpose is to adopt a LASSO method to identify the important clinical features, and use them for PE prediction via a logistic regression model [32]. However, this framework requires a (generalized) linear additive assumption between the clinical features and the propensity of developing PE, which may not hold and is unverifiable in practice. On the other hand, a newly proposed permutation-based feature importance test (PermFIT) provides a universal framework for various machine learning models to identify potential highly correlated important features [33]. Motivated by the challenge of identifying important clinical features associated with the risk of PE under the complex functional relationship, in this paper, we present a permutation-based feature importance test for DNN model (PermFIT-DNN). The PermFIT-DNN framework adapts the appealing feature of PermFIT procedure and the power of our recently proposed scoring algorithm to improve the stability of conventional DNN model [34].

Overall, the aim of this paper is to present an efficient framework for identifying important clinical features related to the risk of pulmonary embolism and to provide early warnings to potential patients who may show asymptomatically. Our analysis reveals that the PermFIT-DNN method outperforms commonly used machine learning techniques in accurately detecting the key features related to the risk of PE. Furthermore, using these identified important features leads to notably accurate predictions of PE risk. The paper is structured as follows: in the Methods section, we outline the process of using machine learning methods to model the complex relationship between risk factors and PE status, and the PermFIT procedure to identify the important features associated with PE status under the complex relationship. In the Results section, the PermFIT-DNN is applied to identify the important clinical features related to PE risk based on data from a clinical study [35] and the results are compared with other machine learning models. Finally, the paper concludes with a brief discussion.

## 2 Methods

### 2.1 Machine learning methods for modeling complex association relationship between clinical features and pulmonary embolism risk

Let **X** = (*X*_1_, …, *X_p_*) be a *p*-dimensional clinical features (e.g., age, body mass index, hypertension, history of PE, etc), and *Y* be the binary PE status (e.g., *Y* = 1 and 0 for PE positive and negative, respectively) with *π*(**X**) = E(*Y*|**X**) = Pr(*Y* = 1|**X**), i.e., the conditional probability of being positive PE given the clinical features **X**. To predict PE status, we need to estimate *π*(**X** = **x**). Traditionally, it is estimated via a logistic regression model. This parametric modeling strategy needs to make a strong assumption on the relationship between the clinical features and the probability of developing PE, which may barely hold and is difficult to verify in practice. To relax this restrictive assumption, machine learning methods are often adopted. Here, we investigate four frequently used machine learning models, i.e., deep neural network (DNN) [28, 29], random forest (RF) [36, 37], and support vector machine (SVM) [38, 39], for their performance in classifying the binary PE status using the clinical features, among which SVM and RF have been used in predicting PE status [31] but not DNN. However, unlike the conventional DNN method, we adopt the stable DNN procedure which can improve prediction performance [34, 40]. Specifically, the stable DNN introduces two extra procedures, i.e., bootstrap aggregating—a machine learning ensemble meta-algorithm [41] is first adopted to increase the stability and accuracy of a single DNN [42]. However, this may not guarantee the stable prediction of each DNN model due to random parameter initialization. To further boost DNN performance, a filtering algorithm is adopted to remove poorly performing bagged DNNs according to the principle that “many could be better than all” [40, 43].

### 2.2 Identification of important clinical features of PE risk via permutation feature importance test (PermFIT)

Though the machine learning methods relax the restrictive assumptions made in the traditional parametric method (e.g., linear or logistic regression) and improve the prediction accuracy, they lack the transparency of interpretation regarding the role of each feature on disease outcome. To identify each feature’s effects on disease outcome under complex functional relationship, we need to establish a valid statistical inference for machine learning models. Herein, we adopt the permutation-based feature importance test (PermFIT) procedure which is applicable to various machine learning models [33]. Based on it, we present a powerful framework to select important clinical features associated with developing PE risk. Although the permutation-based feature importance assessment methods have been proposed for the random forest and DNN models, these methods either do not conduct any statistical inference or cannot provide valid inference regarding the feature importance [44, 45].

We define the feature importance score *M*_*j*_ of *X*_*j*_ (i.e., the *j*^th^ feature in **X** (*j* = 1, …, *p*)) as the expected squared difference between π(X) and $\pi (X^{\left(j\right)})$, where $X^{\left(j\right)}=\left(X_{1},\ldots ,X_{j-1},X_{j^{\prime }},X_{j+1},\ldots ,X_{p}\right)$ is a rearranged **X** with its *j*^*th*^ feature replaced by *X*_*j*′_, a random permutation of the elements of *X*_*j*_. The importance score *M*_*j*_ can be re-expressed as $M_{j}={\text{E}}_{X,X_{j^{\prime }}}{\left[\pi \left(X\right)-\pi (X^{\left(j\right)})\right]}^{2}$, which is zero only when *π*(**X**) ≡ *π*(**X**^(*j*)^), implying no contribution of *X*^(*j*)^ on **X**^(*j*)^ conditional on the other covariates. The stronger the impact of *X*^(*j*)^ on **X**^(*j*)^, the larger *M*_*j*_ is expected to be. Furthermore, *M*_*j*_ can be estimated empirically. Let $X_{j}^{\prime }=\left(X_{s_{1},j},\ldots ,X_{s_{n},j}\right)$ be a random sample of the elements in *X*_*j*_ without replacement, and the empirical permutation importance score be $M_{j}^{\left(P\right)}=\frac{1}{n}\sum_{i=1}^{n}M_{ij}^{\left(P\right)}$ where

$$\begin{array}{c} M_{ij}^{\left(P\right)}=Y_{i}\text{log}(\frac{\hat{\pi }\left(X_{i.}\right)}{\hat{\pi }\left(X_{i.}^{\left(j\right)}\right)})+\left(1-Y_{i}\right)\text{log}(\frac{1-\hat{\pi }\left(X_{i.}\right)}{1-\hat{\pi }\left(X_{i.}^{\left(j\right)}\right)}) \end{array}$$

with **X**_*i*·_ = (*X*_*i*1_, …, *X_ip_*) and $X_{i\cdot }^{\left(j\right)}=\left(X_{i1},\ldots ,X_{i,j-1},X_{s_{i},j},X_{i,j+1},\ldots ,X_{ip}\right)$.

Note that $\text{E}\left[M_{j}^{\left(P\right)}\right]=\text{E}\left[M_{ij}^{\left(P\right)}\right]=\frac{n-1}{n}M_{j}$. The estimate of *π*(⋅), i.e. $\hat{\pi }\left(\cdot \right)$, can be obtained using the aforementioned machine learning models, and the parametric logistic regression (LOG). Particularly, the DNN method we used is the stable DNN model [34, 40]. We estimate $M_{j}^{P}$ as

$$\begin{array}{c} {\hat{M}}_{j}^{\left(P\right)}=\frac{1}{n}\sum_{i=1}^{n}[Y_{i}\text{log}(\frac{\hat{\pi }\left(X_{i.}\right)}{\hat{\pi }\left(X_{i.}^{\left(j\right)}\right)})+\left(1-Y_{i}\right)\text{log}(\frac{1-\hat{\pi }\left(X_{i.}\right)}{1-\hat{\pi }\left(X_{i.}^{\left(j\right)}\right)})] \end{array}$$

Under finite sample size, to avoid a potential overfitting of the approximator $\hat{\pi }\left(\cdot \right)$ using the machine learning method, we employ a cross-fitting strategy to separate the input data into training and validation sets, with the training set used for generating $\hat{\pi }\left(\cdot \right)$ and the testing set for estimating ${\hat{M}}_{j}^{\left(P\right)}$. Let ${\hat{\pi }}_{T}\left(\cdot \right)$ be the estimate of *π*(⋅) from the training set, and $D_{V}={\left\{Y_{i},X_{i\cdot }\right\}}_{i=1}^{n_{V}}$ be the validation set, we obtain the feature importance score estimate ${\hat{M}}_{j}^{\left(P\right)}$, and the variance estimate of ${\hat{M}}_{j}^{\left(P\right)}$ as:

$$\begin{array}{c} {\hat{M}}_{j}^{\left(P\right)}=\frac{1}{n_{V}}\sum_{i=1}^{n_{V}}[Y_{i}\text{log}(\frac{{\hat{\pi }}_{T}\left(X_{i.}\right)}{{\hat{\pi }}_{T}\left(X_{i.}^{\left(j\right)}\right)})+\left(1-Y_{i}\right)\text{log}(\frac{1-{\hat{\pi }}_{T}\left(X_{i.}\right)}{1-{\hat{\pi }}_{T}\left(X_{i.}^{\left(j\right)}\right)})] \end{array}$$

$$\begin{array}{c} \hat{\text{Var}}\left[{\hat{M}}_{j}^{\left(P\right)}\right]=\frac{1}{n_{V}}\sum_{i=1}^{n_{V}}{\left[Y_{i}\text{log}(\frac{{\hat{\pi }}_{T}\left(X_{i.}\right)}{{\hat{\pi }}_{T}\left(X_{i.}^{\left(j\right)}\right)})+\left(1-Y_{i}\right)\text{log}(\frac{1-{\hat{\pi }}_{T}\left(X_{i.}\right)}{1-{\hat{\pi }}_{T}\left(X_{i.}^{\left(j\right)}\right)})-{\hat{M}}_{j}^{\left(P\right)}\right]}^{2} \end{array}$$

Based on it, we construct the test statistic for importance hypothesis test of feature *X*_*j*_ and given as the following:

$$\begin{array}{c} \delta =\frac{{\hat{M}}_{j}^{\left(P\right)}}{\sqrt{\hat{\text{Var}}\left[{\hat{M}}_{j}^{\left(P\right)}\right]}} \end{array}$$ (1)

The PermFIT framework can be summarized in the following Algorithm 1, and the implemented R package is available at https://github.com/SkadiEye/deepTL.

**Algorithm 1** Important Feature Identification Procedure for Machine Learning Models

1: Pre-specify a significance cutoff p-value and randomly split the data as training set and testing set.

2: Within the training set, the machine learning model is adopted to evaluate the test statistic *δ* via Eq (1) and the corresponding p-value for each feature.

3: Identify the important features by comparing the evaluated p-values with the pre-specified cutoff.

### 2.3 Data source

In this paper, we applied the PermFIT framework to a cohort of asthma exacerbation patients from our early retrospective clinical study (with permission granted by the institutional review board (IRB) of the University of Florida (UF), Gainesville, Florida (IRB #: 201802508)) [35], to identify the important features associated with acute PE. The raw data were extracted from the patients’ electronic health records in fully anonymized format with the requirement for informed consent waived by IRB. This led to a total of 3, 660 samples being extracted.

Among the total 3, 660 asthma exacerbation patients, the final study sample included 758 patients who underwent CTA in our analysis. Among these patients, a total of 145 were confirmed positive PE patients via CTA. Under the PermFIT framework, we adopt the aforementioned machine learning models, i.e., stable DNN, random forest, support vector machine, and a parametric logistic regression model to identify the important features associated with PE status among the 22 collected clinical features. Summary statistics of 16 major clinical features of total 22 features are summarized in Table 1, while the rest of 6 clinical features include: atrial fibrillation (97 for yes and 661 for no), ED visit in previous year (130 for yes and 628 for no), coronary artery disease or peripheral vascular disease (178 for yes and 580 for no), use of contraceptives (13 for yes and 745 for no), fractures or general anesthesia in prior month (4 for yes and 754 for no), hemoptysis (7 for yes and 751 for no).

**Table 1:** Major clinical feature summary statistics.

Categorical Feature		No. (%)
Gender	Male	226 (29.82%)
	Female	532 (70.18%)
PE history	Yes	80 (10.55%)
	No	678 (89.45%)
Deep vein thrombosis history	Yes	42 (5.54%)
	No	716 (94.46%)
Hypertension	Yes	487 (64.25%)
	No	271 (35.75%)
Congestive heart failure	Yes	137 (18.07%)
	No	621 (81.93%)
Cancer history	Yes	262 (34.56%)
	No	496 (65.44%)
Race	Black	290 (38.26%)
	Non-black	468 (61.74%)
Cerebrovascular disease	Yes	37 (4.88%)
	No	721 (95.12%)
Diabetes	Yes	247 (32.59%)
	No	511 (67.41%)
Hyperlipidemia	Yes	307 (40.50%)
	No	451 (59.50%)
Smoking history	Yes	432 (56.99%)
	No	268 (35.36%)
	Unknown	58 (7.65%)
Use of inhaled steroids	Yes	375 (49.47%)
	No	383 (50.53%)
Chronic prednisone use	Yes	170 (22.43%)
	No	588 (77.57%)
**Continuous Feature**		**Mean (SD)**
Age		52.20 (16.20)
Body mass index		31.50 (9.21)
Heart rate		100.46 (19.05)

## Results

We adopted a cross-validation in approximately 10: 1 ratio by randomly selecting 65 samples for testing and the rest used for training. In the analysis, we set 4 hidden layers with 50, 40, 30, 20 hidden nodes at each layer for the stable DNN method. For the RF method, we grew 1, 000 trees, and the hyper-parameter settings for RF and SVM were searched via a cross-validation. Under the PermFIT framework (with 100 permutations), we compared the DNN, RF, and SVM method (referred as PermFIT-DNN, PermFIT-RF, and PermFIT-SVM, respectively) and logistic regression (referred as PermFIT-LOG) for identifying the important clinical features at the significance level 0.05. The identified important features by each model and the corresponding p-values are presented in Table 2. Results of Table 2 indicate that different method identifies different set of important features. At the significance level of 0.05, the logistic regression, support vector machine, random forest, and stable DNN method identified 3, 2, 2, and 3 clinical features as the important features among total 22 features. Among the identified important features, we notice that the PE history is unanimously claimed by all models as the highly important feature.

**Table 2:** Identified important clinical features.

Method	Important Clinical Features	P-value
PermFIT-LOG	PE history	<0.001
	Chronic prednisone use	0.014
	Hypertension	0.034
PermFIT-SVM	PE history	<0.001
	Race	0.008
PermFIT-RF	PE history	<0.001
	Hyperlipidemia	0.001
PermFIT-DNN	PE history	<0.001
	Chronic prednisone use	0.011
	Deep vein thrombosis history	0.028

With the selected important features, we evaluated the performance for predicting PE and draw comparison with the corresponding method using all 22 clinical features based on the testing samples. In evaluating the classification performance, besides the accuracy (Accuracy) and area under the receiver operating characteristic curve (AUC), we also adopted the precision-recall AUC (PR-AUC) since this data set is imbalanced, i.e., about 19.1% positive PE. PR-AUC is a useful cut-off independent metrics to evaluate the performance of a classifier on positive samples. For the accuracy evaluation, we used the cutoff probability 0.5. That is, the PE status (i.e., *y*_*i*_) for a patient *i* with clinical features **X** = **X** is predicted as:

$${\hat{y}}_{i}=\{\begin{array}{ll} 1 & \text{if}\;{\hat{\pi }}_{i}(x)>0.5\\ 0 & \text{if}\;{\hat{\pi }}_{i}(x)\leq 0.5 \end{array}$$

Results of Table 3 clearly demonstrate that three machine learning models, i.e., the stable DNN, random forest, and SVM noticeably outperform the parametric logistic regression model in terms of Accuracy, AUC and PR-AUC predictions when all the 22 features are included, and achieve (0.862, 0.774, 0.956), (0.892, 0.775, 0.956), and (0.892, 0.700, 0.935) for (Accuracy, AUC, PR-AUC) predictions, respectively. In contrast, the predictions of for (Accuracy, AUC, PR-AUC) via the logistic regression are (0.108, 0.252, 0.771). This implies that there exist complex functional relationship between clinical features and PE status, which the logistic regression model have severely misspecified the complex functional relationship. Furthermore, though the stable DNN and random forest methods have almost identical performance in terms of AUC and PR-AUC predictions, they are all superior to the SVM method. This observation demonstrates that the stable DNN and random forest can better model the complex functional relationship using all 22 features. However, further examining the difference of Accuracy, AUC, and PR-AUC predictions between using all 22 features and using the identified important features, it is evident that there exist minor or no decrease for the stable DNN method, i.e., DNN vs PermFIT-DNN as (0.862, 0.774, 0.956) vs (0.892, 0.759, 0.940). On the other hand, comparing the prediction difference for AUC and PR-AUC using all 22 features with that including the identified important features only for random forest method, there exist notable decrease, i.e., RF vs PermFIT-RF as (0.775, 0.956) vs (0.710, 0.916). This observation indicates that the important features determined by the random forest method are not very reliable. Therefore, using the important features identified by the random forest method could not accurately classify PE status.

**Table 3:** PE prediction performance comparison.

Method	Accuracy	AUC	PR-AUC
DNN^a^	0.862	0.774	0.956
PermFIT-DNN^b^	0.892	0.759	0.940
RF^a^	0.892	0.775	0.956
PermFIT-RF^b^	0.892	0.710	0.916
SVM^a^	0.892	0.700	0.935
PermFIT-SVM^b^	0.892	0.733	0.930
Logistic^a^	0.108	0.252	0.771
PermFIT-LOG^b^	0.108	0.209	0.779

^a^: Include all 22 collected clinical features in prediction model

^b^: Include identified important clinical features only in prediction model

The combination of the results from Tables 2 and 3 shows that using the identified important features can achieve predictions that are almost as accurate as those obtained using all 22 features when the stable DNN model is used. This finding suggests that the important features identified by the stable DNN method can effectively determine PE patients. Particularly, it can result in a remarkable PR-AUC prediction of 0.940, implying that the identified clinical features can accurately characterize PE patients. In clinical practice, this means that the history of PE, chronic prednisone use, and deep vein thrombosis can be used to identify potential PE patients, who can then be confirmed by CTA. It is worth noting that although PermFIT-DNN outperforms two other machine learning models considered in this paper for robustly identifying important features, its predicted accuracy and AUC are not very high (see the confusion matrix in Table 4). This suggests that some important clinical features have not been collected. However, this does not diminish the usefulness of the PermFIT-DNN method for identifying features associated with PE status.

**Table 4:** Confusion matrix via PermFIT-DNN.

	Observed Positive (OP)	Observed Negative (ON)
**Predicted Positive (PP)**	4	2
**Predicted Negative (PN)**	5	54

## Discussion

In this paper, we used a permutation-based feature importance test procedure to investigate various machine learning models and a parametric logistic regression model to identify the important clinical features related to PE status. Our results indicated that the PermFIT-DNN framework, which combines PermFIT with the stable DNN model, can effectively identify important clinical features. Additionally, using these important features, the prediction performance was non-inferior in terms of all metrics considered, affirming the reliability of the identified features. These results clearly demonstrate the advantages of the permutation feature importance test procedure through the stable DNN model (i.e., the PermFIT-DNN framework) in clinical practice for identifying important features associated with PE risk. However, it should be noted that this study had some limitations, such as being a small single-center study with limited clinical features collected. Larger multi-center studies with more complete clinical features should be conducted in the future. Furthermore, the variance of accuracy estimate can be underestimate due to the overlapping of training samples in cross-validation [46]. However, this should not change the superiority of PermFIT-DNN method over other competing methods such as PermFIT-RF, PermFIT-SVM and PermFIT-LOG since the performance comparisons were based on the same training and testing samples. Also, it should not undermine the usefulness of the derived PermFIT-DNN framework for identifying important features associated with PE risk in clinical practice. With larger sample sizes and more complete clinical features collected, we expect that the PermFIT-DNN framework will identify a robust set of important clinical features to further improve PE prediction accuracy.

## Supporting information

S1 Data (XLSX)
